# Competing endogenous RNA network profiling reveals novel host dependency factors required for MERS-CoV propagation

**DOI:** 10.1080/22221751.2020.1738277

**Published:** 2020-03-30

**Authors:** Xi Zhang, Hin Chu, Lei Wen, Huiping Shuai, Dong Yang, Yixin Wang, Yuxin Hou, Zheng Zhu, Shuofeng Yuan, Feifei Yin, Jasper Fuk-Woo Chan, Kwok-Yung Yuen

**Affiliations:** a State Key Laboratory of Emerging Infectious Diseases, The University of Hong Kong, Pokfulam, Hong Kong Special Administrative Region, People's Republic of China; b Department of Microbiology, Li Ka Shing Faculty of Medicine, The University of Hong Kong, Pokfulam, Hong Kong Special Administrative Region, People's Republic of China; c Hainan Medical University-The University of Hong Kong Joint Laboratory of Tropical Infectious Diseases, Hainan Medical University, Haikou, People's Republic of China, and The University of Hong Kong, Pokfulam, Hong Kong Special Administrative Region, People's Republic of China; d Department of Pathogen Biology, Hainan Medical University, Haikou, People’s Republic of China; e Key Laboratory of Translational Tropical Medicine of Ministry of Education, Hainan Medical University, Haikou, People’s Republic of China; f Carol Yu Centre for Infection, Li Ka Shing Faculty of Medicine, The University of Hong Kong, Pokfulam, Hong Kong Special Administrative Region, People's Republic of China; g Department of Clinical Microbiology and Infection Control, The University of Hong Kong-Shenzhen Hospital, Shenzhen, People’s Republic of China; h The Collaborative Innovation Center for Diagnosis and Treatment of Infectious Diseases, Li Ka Shing Faculty of Medicine, The University of Hong Kong, Pokfulam, Hong Kong Special Administrative Region, People's Republic of China

**Keywords:** MERS-CoV, competing endogenous RNA, circRNA, miRNA, mRNA

## Abstract

Circular RNAs (circRNAs) are an integral component of the host competitive endogenous RNA (ceRNA) network. These noncoding RNAs are characterized by their unique splicing reactions to form covalently closed loop structures and play important RNA regulatory roles in cells. Recent studies showed that circRNA expressions were perturbed in viral infections and circRNAs might serve as potential antiviral targets. We investigated the host ceRNA network changes and biological relevance of circRNAs in human lung adenocarcinoma epithelial (Calu-3) cells infected with the highly pathogenic Middle East respiratory syndrome coronavirus (MERS-CoV). A total of ≥49337 putative circRNAs were predicted. Among the 7845 genes which generated putative circRNAs, 147 (1.9%) of them each generated ≥30 putative circRNAs and were involved in various biological, cellular, and metabolic processes, including viral infections. Differential expression (DE) analysis showed that the proportion of DE circRNAs significantly (*P* < 0.001) increased at 24 h-post infection. These DE circRNAs were clustered into 4 groups according to their time-course expression patterns and demonstrated inter-cluster and intra-cluster variations in the predicted functions of their host genes. Our comprehensive circRNA-miRNA-mRNA network identified 7 key DE circRNAs involved in various biological processes upon MERS-CoV infection. Specific siRNA knockdown of two selected DE circRNAs (circFNDC3B and circCNOT1) significantly reduced MERS-CoV load and their target mRNA expression which modulates various biological pathways, including the mitogen-activated protein kinase (MAPK) and ubiquitination pathways. These results provided novel insights into the ceRNA network perturbations, biological relevance of circRNAs, and potential host-targeting antiviral strategies for MERS-CoV infection.

## Introduction

Circular RNAs (circRNAs) are noncoding RNAs characterized by their unique splicing reactions to form covalently closed loop structures [1–3]. They are abundantly found in human cells and play important RNA regulatory roles, including acting as microRNA (miRNA) sponges, interacting with RNA binding proteins (RBPs), as well as modulating the transcription of parental genes by interacting with RNA polymerase II [2,4,5]. The competitive endogenous RNA (ceRNA) hypothesis proposes that RNA transcripts, including circRNAs, messenger RNAs (mRNAs), and long non-coding RNAs (lncRNAs), contain miRNA response elements (MREs) which compete among themselves for miRNA binding to regulate the expression of each other [6,7]. circRNAs possess miRNA-sponging activity and counteract the miRNA’s inhibitory activity on the target mRNA [6,7]. Recent studies showed that the expressions of circRNAs were perturbed in viral infections caused by both DNA (hepatitis B virus, Kaposi’s sarcoma-associated herpesvirus, and simian vacuolating virus 40) and RNA [Ebola virus, porcine epidemic diarrhea virus (PEDV), transmissible gastroenteritis virus (TGEV), influenza A viruses, and avian leukosis virus subgroup-J] viruses [8–15], and that these circRNAs might serve as potential antiviral targets [9,12,16].

Middle East respiratory syndrome coronavirus (MERS-CoV) is an emerging human-pathogenic coronavirus which has caused >2400 infections and >800 deaths worldwide since 2012 [17–19]. Despite a high mortality rate of >30%, the virus-host interaction and pathogenesis of this emerging infection remains incompletely understood [20]. Moreover, the host ceRNAs profile changes and the functions of circRNAs in MERS-CoV and other human-pathogenic coronavirus infections have not been reported. In this study, we used MERS-CoV as a model to investigate the host ceRNA network changes and biological relevance of circRNAs in CoV infection. We showed that MERS-CoV significantly perturbed a high number of circRNAs, miRNAs, and mRNAs which were involved in a wide range of biological processes. We also validated the effects of selected circRNAs in MERS-CoV replication and provided new insights into potential host-targeting antiviral strategies through the manipulation of circRNAs.

## Materials and methods

### Virus and cells

MERS-CoV (strain HCoV-EMC/2012) was kindly provided by Ron Fouchier (Erasmus Medical Center, Rotterdam, the Netherlands) [21]. Clinical isolates of SARS-CoV (GZ50) and influenza A virus strain A/HongKong/415742/2009(H1N1)pdm09 were obtained for validation studies from Department of Microbiology at The University of Hong Kong and prepared as previously described [22]. Calu-3 (human lung adenocarcinoma) cells were used to establish the MERS-CoV replication model for transcriptomic study as previously described according to Biosafety Level 3 practice [22,23]. The cells were maintained in Dulbecco’s Modified Eagle Medium/F12 (DMEM/F12) supplemented with 10% heat-inactivated fetal bovine serum (FBS), 100 U/ml penicillin and 100 µg/ml streptomycin as previously described [24,25]. Primary human embryonic lung fibroblasts (HFL) were maintained in supplemented Minimum Essential Medium (MEM) as we described previously [26].

### Sample preparation and total RNA isolation

Calu-3 cells (10^7^ cells per biological replicate, three biological replicates), were mock-infected or infected with MERS-CoV at multiplicity of infection (MOI) of 4. The cell pellets were harvested at 24 h post-infection (hpi) in 1 ml of lysis buffer. Total RNA was extracted using the TRIzol reagent (Invitrogen, Carlsbad, CA, USA) according to the manufacturer’s instructions, followed by DNase I digestion (Epicentre, Madison, WI, USA) for 15 min at 37°C. The integrity and quality of the extracted total RNA were evaluated using an Agilent 4200 Bioanalyzer (Agilent Technologies, Santa Clara, CA, USA) with RNA integrity number (RIN) >7.0. The RNA quantity was measured using NanoDrop^TM^ One (Thermo Fisher Scientific, Waltham, MA, USA).

### Library construction and Illumina sequencing

A circRNA library, a small RNA library, and a mRNA library were constructed for the identification of circRNA, miRNA, and mRNA, respectively. To prepare for circRNA sequencing, linear RNAs were removed using RNase R (RNR07250, Epicentre) (1 unit/ μg) for 20 min treatment at 37°C. Ribosomal RNA (rRNA) was depleted in the total RNA using a Ribo-Zero Gold Kit (Epicentre) following the manufacturer’s instructions. After purification, the rRNA-depleted RNA products were fragmented using VAHTS Total RNA-seq (H/M/R) Library Prep Kit for Illumina (Vazyme Biotech Co., Ltd, Nanjing, Jiangsu, China). Three cDNA libraries were sequenced on Illumina Hiseq X-Ten platform (HaploX Biotechnology, Jiangxi, China) and 2×150 bp paired-end (PE150) reads were obtained using the HiSeq Control Software (HD3.5.0). Next, the sequencing reads were conducted for real-time sequencing image analysis and base-calling using Real-Time Analysis (v2.7.7). All Illumina sequencing raw and processed data were submitted to the GEO database (http://www.ncbi.nlm.nih.gov/geo/) under the accession number GSE139516.

### Identification of circRNAs, miRNAs, and mRNAs

The raw reads were subjected to quality assessment using fastp (0.19.5) [27]. Reads containing adapter, reads of N base over 5 bp, and low-quality reads were removed to obtain high quality clean reads. To identify circRNA, the clean reads were aligned with the human reference genome GRCh38 (http://genome.ucsc.edu/) using HISAT2 (https://bio.tools/hisat2). CIRI2 [28] and find_circ (version 1.2) [29] were used for the prediction of putative circRNAs. Overlapping circRNAs in CIRI2 and find_circ were selected for further analysis. The Burrows–Wheeler Alignment tool was used to identify miRNA [30]. Unique sequences containing 18–35 nucleotides were mapped to miRBase 22.0 by BLAST search to identify known and novel miRNAs. Due to the short lengths, the expression of circRNA and miRNA was normalized to transcripts per million (TPM), where TPM = (actual miRNA/circRNA counts of total clean read) × 10^6^. To identify mRNA, after alignment with the human reference genome GRCh38 with HISAT2, clean reads were quantified based on the number of reads spanning the back-splicing junction, and their fragments per kb for a million reads (FPKM) were calculated using HTSeq 0.10.0. RNAs with |log_2_ (fold change)| ≥1 and adjust *P* value <0.05 were defined as differentially expressed (DE) by DESeq2 (version 1.18.1).

### Weighted gene co-expression network analysis (WGCNA)

WGCNA was performed to identify mRNA-circRNA pairs with positive correlations. The WGCNA R package was downloaded from Bioconductor (https://bioconductor.org/) and was applied to the dissimilarity matrix (1-TOM) to find clusters in each network. The DE mRNAs and DE circRNAs were clustered into different modules. To better understand the biological function of each module, we performed pathway analysis for all the DE circRNAs and DE mRNAs that belonged to the same module.

### Integrated analysis of circRNA–miRNA–mRNA network

circRNA–miRNA–mRNA interaction networks were constructed as described below. First, correlation analysis between DE circRNAs and DE miRNAs was performed and the correlation *P* value (cP) was calculated based on Pearson correlation coefficients. circRNA-miRNA pairs with strong negative correlations were defined as those having Pearson correlation coefficient (r) < −0.7 and cP <0.05. The circRNA–miRNA pairs with strong negative correlations were selected for circRNA-miRNA binding site prediction using miRanda (http://www.microrna.org/) v3.3a (-sc 140 -en −1.0 -scale 4 -out) [31]. Second, correlation analysis between DE miRNAs and DE mRNAs was performed using the same method. The miRNA-mRNA pairs with strong negative correlations were again defined as those having r < −0.7 and c*P* value <0.05. The miRNA-mRNA pairs with strong correlations were selected for miRNA-mRNA binding site prediction using miRanda. Finally, correlation analysis between DE mRNAs and DE circRNAs was performed. The mRNA-circRNA pairs with strong positive correlations were defined as those having r >0.7 and cP <0.05. The final circRNA-miRNA-mRNA network graphs were constructed and visualized using Cytoscape v3.5.1 (http://www.cytoscape.org/).

### RNase R resistance analysis of circRNAs and quantitative reverse transcription-polymerase chain reaction (qRT-PCR)

The total RNA (1μg) extracted from the Calu-3 cells was treated with 1 unit of RNase R or nuclease-free water (mock control) and incubated for 20 min at 37°C. The digested RNA was purified using miRNeasy Mini Kit (Qiagen, Hilden, Germany). Divergent primers for the selected circRNAs were designed through CircInteractome (https://circinteractome.nia.nih.gov/) (Supplementary Table 1) [32]. Then, the treated RNAs were subjected to reverse transcription and quantitative polymerase chain reaction (qRT-PCR) with Transcriptor First Strand cDNA Synthesis Kit and LightCycler 480 master mix (Roche Holding AG, Basel, Switzerland) as previously described [26,33,34]. The housekeeping gene, GAPDH, was used as an internal control. The comparative CT (2^−ΔΔCT^) method was used to obtain the fold change of circRNA expression levels.

### siRNA knockdown and infection

Customized siRNAs were designed through CircInteractome and synthesized by Dharmacon (Lafayette, CO, USA). The detailed sequences of siRNAs were listed in Supplementary Table 1. Cells were transfected with 70nM siRNA using Lipofectamine RNAiMAX (Thermo Fisher Scientific) twice over two consecutive days as previously described [26,35]. At 24 h after the second transfection, the cells were challenged with MERS-CoV (MOI = 0.1). The inoculum was removed after 1 h at 37°C. The knockdown efficiency was assessed in parallel by qRT-PCR. At 24 hpi, the cells were harvested for further analysis. The cell viability of the siRNA-treated cells were evaluated with CellTiterGlo® as described before [36].

### Plasmids construction and transfection

Full lengths of hsa_circ_0067985 and hsa_circ_0006275 were synthesized and subcloned into the pCD25-ciR vector and verified by sequencing in Geneseed Biotech Co.,Ltd (Guangzhou, China). The recombinant plasmids were transfected into Calu-3 cells using the Lipofectamine™3000 transfection reagent (Life Technologies) according to the manufacturer's protocol. The expression of each circRNA was measured at 36 h post-transfection to determine the transfection efficiency.

### Statistical analyses

All data were analysed with GraphPad Prism (version 6.0, GraphPad, Inc) as we previously described [37]. The *P*-values were adjusted using Benjamini and Hochberg's approach to control the false discovery rate. Student’s t-test was used to determine significant differences in gene expression changes in host cells after siRNA knockdown of selected circRNAs. Chi-square test was used to compare the number of DE circRNAs, DE miRNAs, and DE mRNAs in MERS-CoV infection. One-way ANOVA was used to determine significant differences in the other parameters between different groups. *P* < 0.05 was considered statistically significant.

## Results

### Landscape of circRNAs, miRNAs, and mRNAs identified in human lung epithelial cells

**Figure 1.**
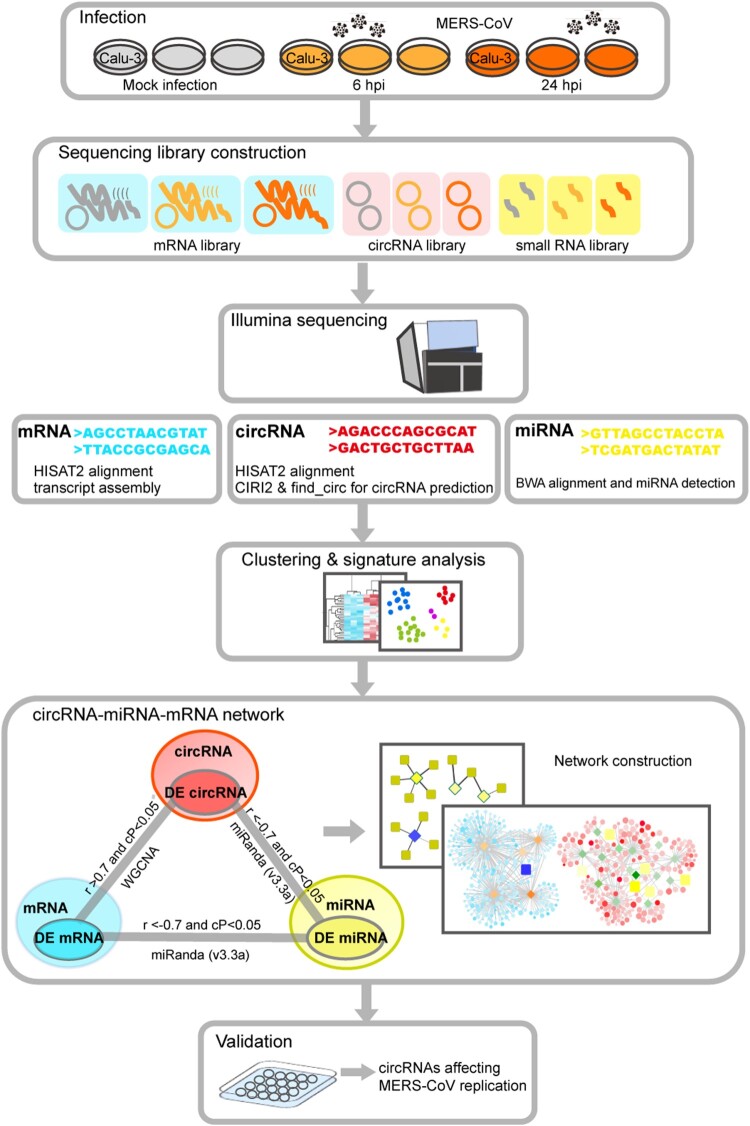
**Schematic overview of RNA sequencing, data analysis, and critical pathogenic circRNAs identification.** Mock-infected and MERS-CoV-infected Calu-3 cells were harvested for RNA sequencing. CIRI2 and find_circ were used for circRNA prediction. Differential expression analysis and co-expression analysis were implemented to profile the impact of MERS-CoV infection on host cells and construct the circRNA-miRNA-mRNA co-regulatory network. The effects of selected circRNAs identified in the circRNA-miRNA-mRNA network on MERS-CoV replication were validated *in vitro*.

**Figure 2.**
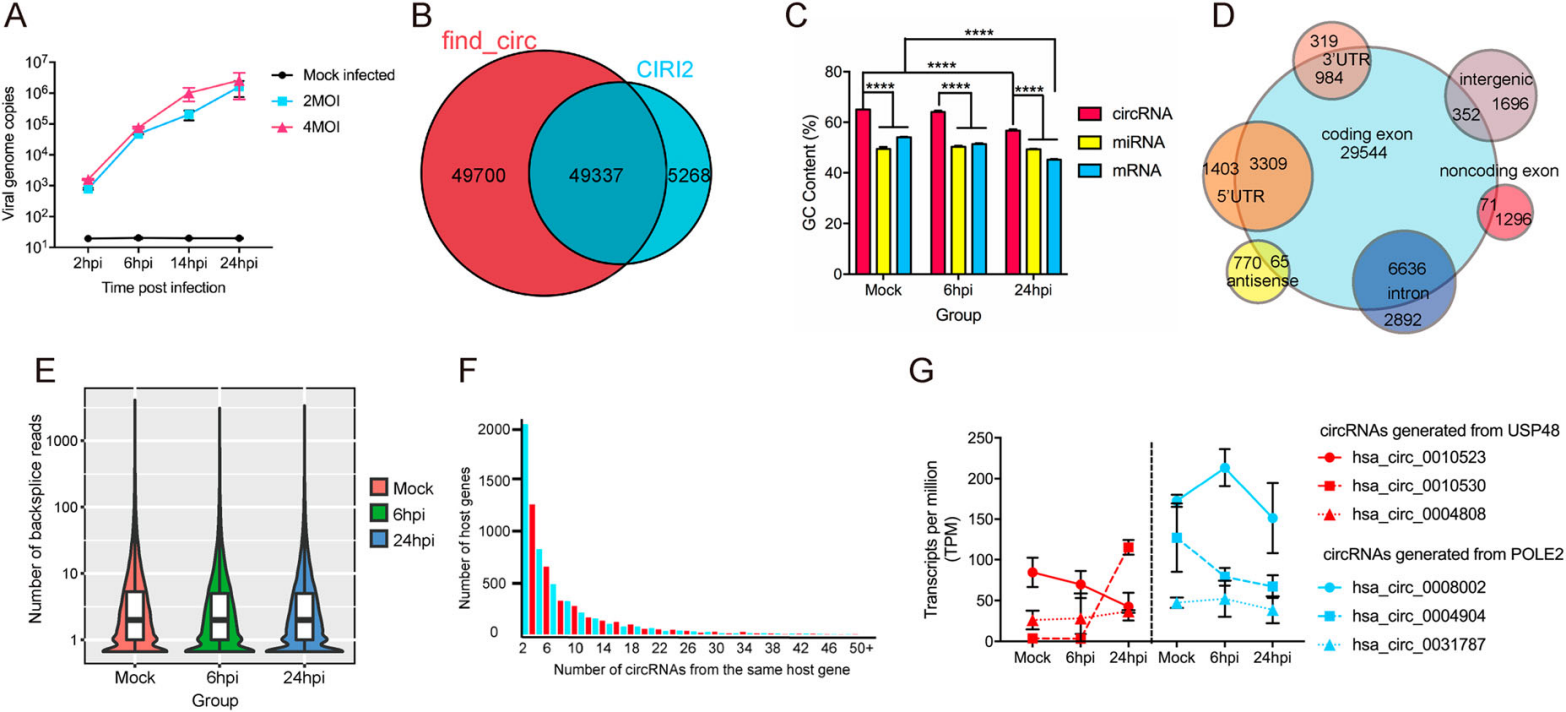
**Characterization of identified circRNAs, miRNAs and mRNAs in MERS-CoV infected and uninfected Calu-3 cells.** (A) qRT-PCR results measuring MERS-CoV nucleocapsid gene copies at the indicated time points. Calu-3 cells were either mock-infected or infected with MERS-CoV at MOIs of 2 and 4. All experiments were carried out in triplicates. (B) Venn diagram showing the overlapped circRNAs identified by CIRI2 and find_circ pipelines. (C) Average GC contents of circRNAs, miRNAs and mRNAs. (D) Genomic distribution of circRNAs identified in Calu-3 cells. (E) Violin plot with the Y-axis showing the distribution of the number of back-splicing reads detected in each circRNA at the three time points indicated in the X-axis. (F) Number of circRNA isoforms derived from the same gene. (G) The diversified expression patterns of circRNAs of USP48 and POLE2. Data in A, C, and G represented means and standard deviations.

A schematic representation of the study design and computational analysis was shown in Figure 1. To optimize the conditions for RNA sequencing, we first determined the viral replication kinetics of MERS-CoV infection (MOI = 2 and 4) in Calu-3 cells by qRT-PCR. As shown in Figure 2A, there was ∼2-log increase and 4-log increase in viral load at 6 hpi (early phase) and 24 hpi (late phase). Next, to obtain a comprehensive landscape of the ceRNA network of the lung epithelial cells with either mock infection or MERS-CoV infection, we performed RNA sequencing on virus-infected Calu-3 cells (MOI = 4) to profile the expression patterns of circRNA, miRNA, and mRNA. To increase the confidence level of circRNA prediction, both CIRI2 and find_circ were used [28,29]. A total of 54605 and 99037 putative circRNAs were predicted by CIRI2 and find_circ, respectively (Figure 2B). Among these putative circRNAs, 49337 were predicted by both CIRI2 and find_circ. These 49337 putative circRNAs had a significantly higher mean GC content than the miRNAs and mRNAs in our study (Figure 2C). Among the 49337 putative circRNAs, 40961 (83.0%) were derived from coding regions (Figure 2D). The majority (41690/49337, 84.5%) of the putative circRNAs containing a small number (1–5) of back splice reads (Figure 2E). A minority (1986/49337, 4.0%) of them had head-to-tail junction with reads ≥ 20. The overall circRNA expression profiles at these three time points were uniform and not significantly altered by MERS-CoV infection. Collectively, these basic characteristics of the putative circRNAs identified in our study were similar to those identified in other human and mammalian cells, including mouse P19 embryonic carcinoma, human neuroblastoma SH-SY5Y, and acute promyelocytic leukemia-derived NB4 [38–40].

The number of different circRNAs generated from individual host genes was highly variable (Figure 2F). Among the 7845 genes which generated putative circRNAs, 147 of them (1.9%) each generated ≥30 putative circRNAs (Supplementary Table 2). These 147 genes were shown to be involved in various biological, cellular, and metabolic processes in Gene Ontology (GO) enrichment analysis (Supplementary Figure 1). Moreover, many of these genes were involved in viral infection. For example, protein tyrosine phosphatase receptor kappa (PTPRK) is downregulated by Epstein–Barr virus in Hodgkin lymphoma (HL), leading to suppressed transforming growth factor-beta (TGF-β) signalling [41]. Membrane-associated guanylate kinase with inverted organization-1 (MAGI-1) is a PDZ protein that interacts with viral proteins containing the PDZ-ligand binding motif, such as influenza A virus NS1, human papillomavirus 16 E6, and human alphapapillomavirus E6 proteins [41–43]. Notably, the different putative circRNAs generated from the same host gene might demonstrate diversified expressions. For example, the expressions of the different putative circRNAs generated from DNA polymerase epsilon subunit 2 (POLE2) and Ubiquitin Specific Peptidase 48 (USP48) varied by >2 folds at 24 hpi (Figure 2G). These diversifications in the expression of the individual circRNAs might be related to their different biogenesis mechanisms and biological functions.

### MERS-CoV infection induced global changes in human lung epithelial cell circRNAs, miRNAs, and mRNAs expression

**Figure 3.**
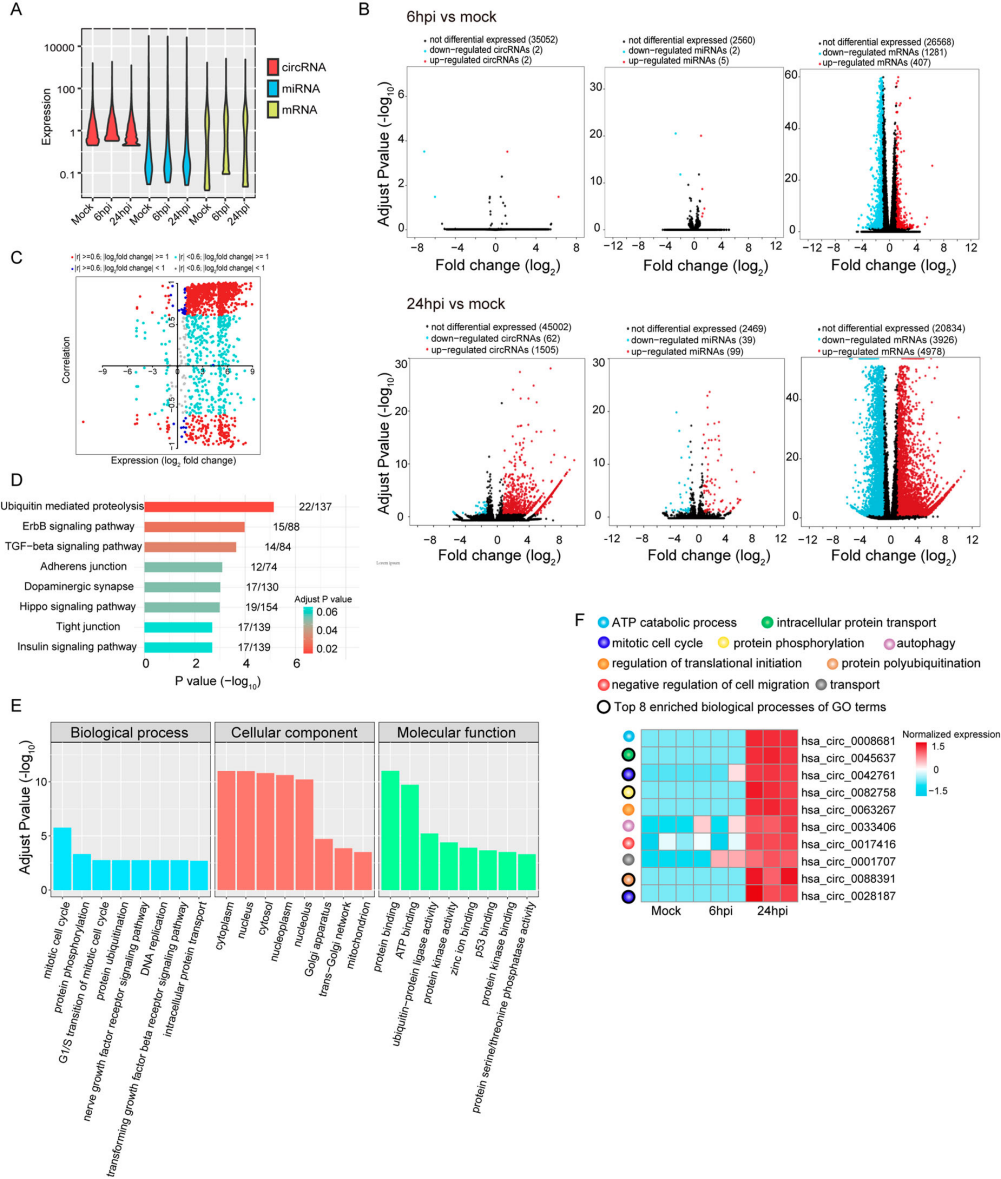
**Global changes on host ceRNAs expression induced upon MERS-CoV infection.** (A) Violin plot showing the expression intensity of circRNAs, miRNAs and mRNAs of MERS-CoV infected and non-infected Calu-3 cells. (B) Volcano plots representation of DE circRNA, miRNA and mRNAs identified (up: 6hpi vs mock; bottom: 24hpi vs mock). The selection criteria was set as |log_2_ (fold change)| ≥1 and adjust *P* value < 0.05. (C) Correlation between DE circRNAs and their corresponding host genes. DE circRNAs were coloured by Pearson correlation coefficient (r) and their expression at 24hpi. (D, E) Bar plot of the top 8 most enriched KEGG pathways and GO terms of the DE circRNA host genes. (F) Heatmap showing the normalized log_2_ [expression values in transcripts per million (TPM)] of the top 10 up-regulated circRNAs identified at 24hpi each annotated with the assigned biological process.

After obtaining the overall landscape of the ceRNAs in lung epithelial cells, we further characterized the differential expressions of circRNAs, miRNAs, and mRNAs in MERS-CoV-infected versus mock-infected cells to elucidate the impact of MERS-CoV infection on the host. The expression of circRNAs, miRNAs, and mRNAs at different time points in MERS-CoV-infected or mock-infected Calu-3 cells were shown in Figure 3A. Differential expression (DE) analysis was then performed to investigate the changes of host ceRNAs upon MERS-CoV infection. At 6 hpi, the proportion of DE circRNAs (4/35056, 0.01%), DE miRNAs (7/2567, 0.3%), and DE mRNAs (1688/28256, 6.0%) in MERS-CoV-infected vs mock-infected samples was very low (Figure 3B, top panels). At 24 hpi, the proportion of DE circRNAs (1567/46569, 3.4%), DE miRNAs (138/2607, 5.3%), and DE mRNAs (8904/29738, 29.9%) in MERS-CoV-infected vs mock-infected samples all significantly (*P* < 0.001) increased (Figure 3B, bottom panels). Additionally, 1267 DE circRNAs, 102 DE miRNAs, and 7385 DE mRNAs were identified when comparing MERS-CoV-infected samples at 6 and 24 hpi. After exclusion of the overlapping DE circRNAs, DE miRNAs, and DE mRNAs in these analyses, a total of 1815 unique DE circRNAs, 153 unique DE miRNAs, and 10254 unique DE mRNAs were identified.

circRNAs may be positively correlated, negatively correlated, or non-correlated with their corresponding host genes [4,29,44,45]. In MERS-CoV infection, we found that 922/1815 (50.8%) and 190/1815 (10.5%) of the DE circRNAs were significantly positively and negatively correlated with their host genes, respectively (Figure 3C). To investigate the potential functions of these DE circRNAs, we performed pathway and functional enrichment analyses on their host genes using Kyoto Encyclopedia of Genes and Genomes (KEGG) pathway and GO analysis, respectively. As shown in Figures 3D and 3E, a wide range of pathways and processes were perturbed. The 10 most upregulated DE circRNAs at 24 hpi and the functions of their host genes were illustrated in Figure 3F. These virus-perturbed functions included ATP metabolism, protein transport, protein phosphorylation and polyubiquitination, mitosis, regulation of translational initiation, and autophagy.

### The DE circRNAs in different clusters exhibited inter-cluster and intra-cluster variations in host pathway perturbations

**Figure 4.**
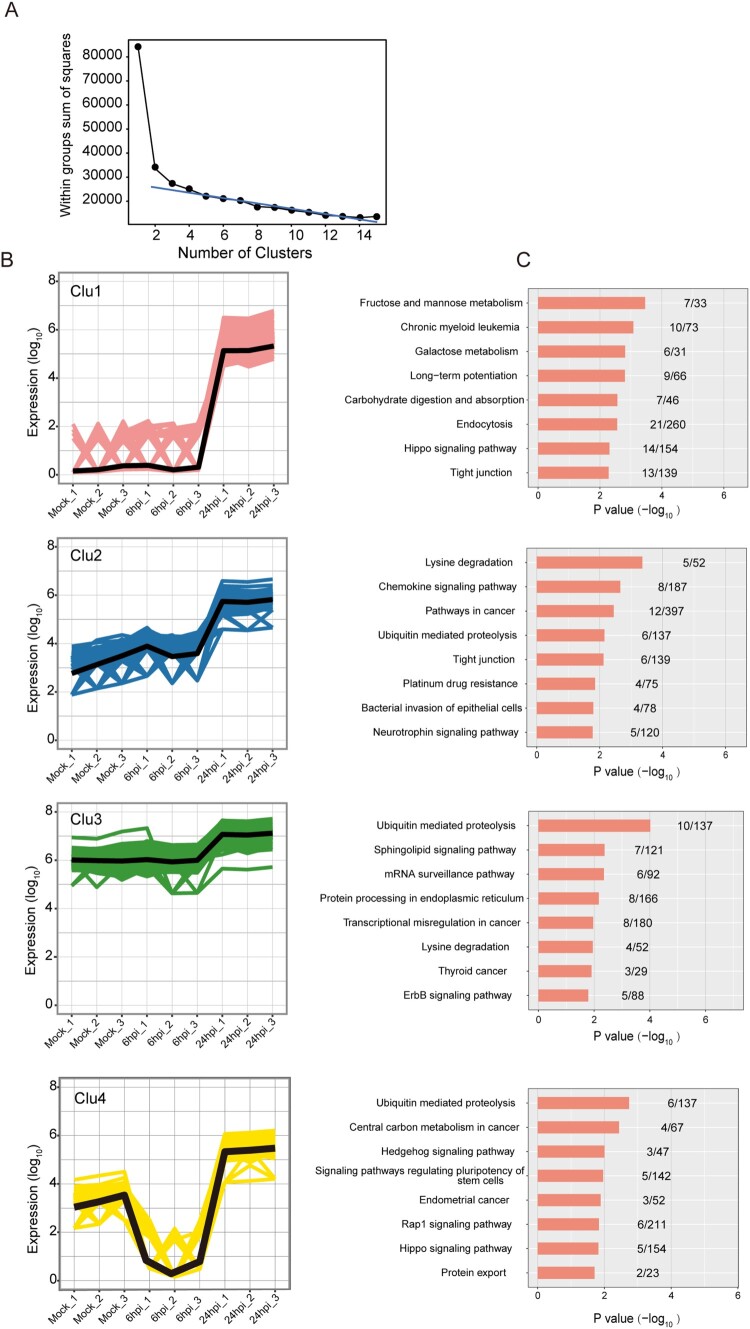
**Overall-activated expression features of DE circRNAs responsive to MERS-CoV infection.** (A) Number of clusters generated in the elbow method to select the optimal cluster number of DE circRNAs profiling. The summed distance of each circRNA from its cluster centroid was assessed and plotted. (B, C) Temporal profiles of the average expression pattern of DE circRNAs and the top 8 enriched KEGG pathways for each cluster.

Next, we conducted K-means clustering analysis to determine the expression kinetics of the DE circRNAs. As shown in Figure 4A, the point of inflexion fell between 3 and 5 in the Elbow method which prompted us to cluster the DE circRNAs into 4 groups (Clu1, Clu2, Clu3, and Clu4). These DE circRNAs demonstrated different time-course expression patterns (Figure 4B). The number of DE circRNAs in each cluster was 1046, 239, 347, and 183, respectively, for Clu1 to Clu4. The mean expression intensities of the DE circRNAs in Clu1, Clu2, and Clu3 in mock-infected cells were similar to those in MERS-CoV-infected cells at 6 hpi (Figures 4B). In contrast, the mean expression intensity of the DE circRNAs in Clu4 in MERS-CoV-infected cells at 6 hpi was lower than that of mock-infected cells. The DE circRNAs in all 4 clusters in MERS-CoV-infected cells at 24 hpi were higher than those in mock-infected cells and MERS-CoV-infected cells at 6 hpi. Using KEGG pathway analysis, we showed that the predicted functions of the host genes of the DE circRNAs in the 4 clusters demonstrated inter-cluster and intra-cluster variations (Figure 4C). Similar to the KEGG analysis of the host gens of the total DE circRNAs (Figure 3D), ubiquitin-mediated proteolysis was the most perturbed pathway in both Clu3 and Clu4 (Figure 4C). This observation implicated that key DE circRNAs that were involved in the regulation of MERS-CoV infection might be enriched in these clusters.

### Weighted gene co-expression network analysis (WGCNA) revealed a wide range of biological processes perturbed by in MERS-CoV infection

**Figure 5.**
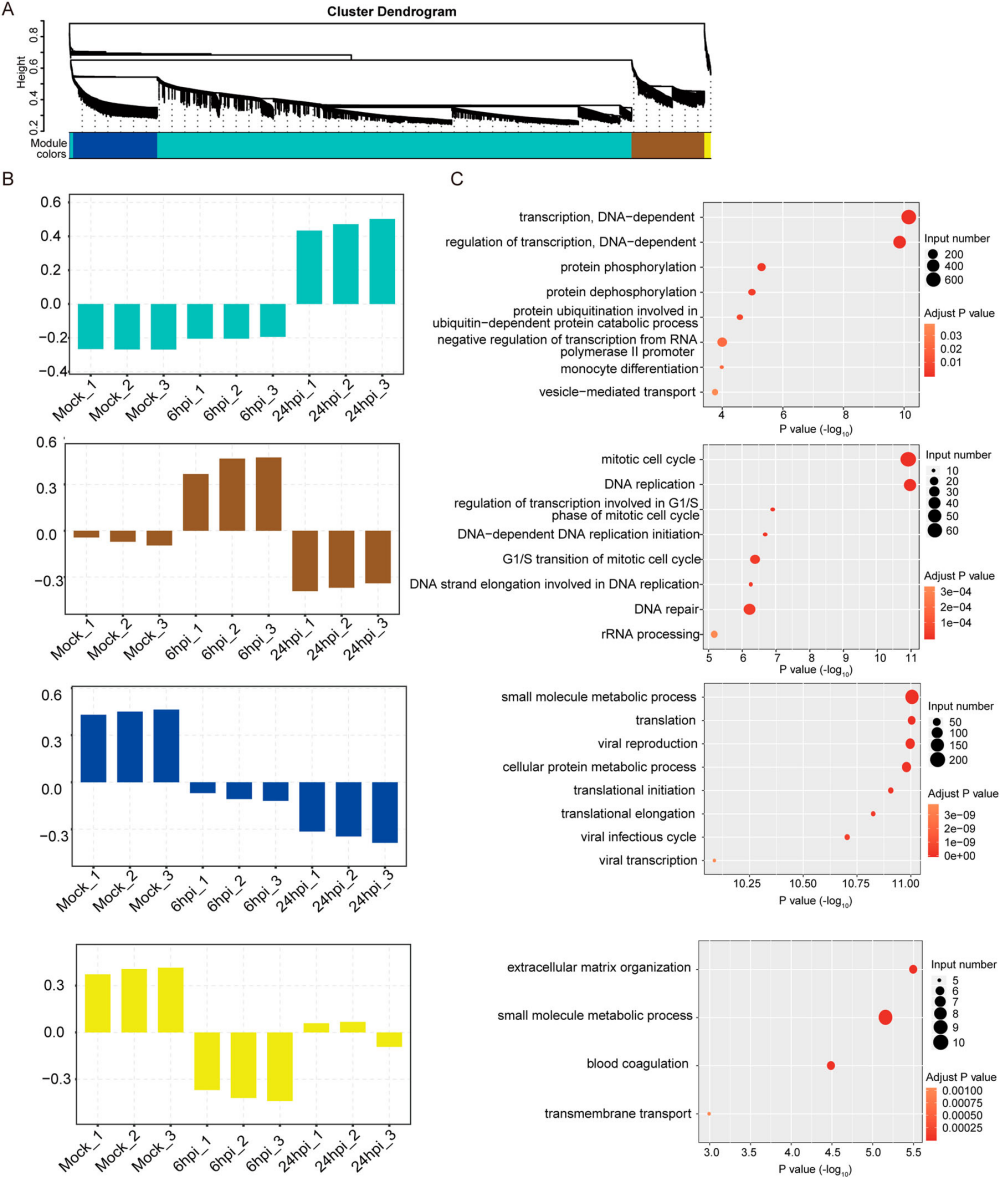
**Identification of circRNA-mRNA co-expression modules and networks associated with the pathogenesis of MERS-CoV.** (A) CircRNA-mRNA modules identified with WCGNA. (B, C) Bar plots of the eigengene values of the modules identified. Host genes of DE circRNAs and DE mRNAs attributed in each module were input to GO database to identify the top 8 overrepresented biological processes.

To further understand the regulations and crosstalk between the DE circRNAs and their corresponding mRNAs, we next performed WGCNA [46]. As circRNAs could either restrict or facilitate viral infection through gene expression regulation, simultaneous examination of DE circRNAs and DE mRNAs might help to decipher the putative functions of the DE circRNAs in MERS-CoV infection [12,13]. This module-based analysis allows examination of co-expressed components with different expression intensities. After constructing a signed network which included positively correlated DE circRNAs and DE mRNAs, we used average linkage hierarchical clustering coupled with the topological overlap dissimilarity measure for module categorization [46]. Our WGCNA signed network consisted of 4 distinct modules, each representing a characteristic co-expression pattern of DE circRNAs and DE mRNAs (Figure 5A). Among the 4 modules, the turquoise module comprised the largest number of DE circRNAs and their co-expressed mRNAs [1709/1815 (94.1%) of DE circRNAs and 7280/10254 (71.0%) of DE mRNAs]. The other 3 modules included 55 (3.0%) DE circRNAs and 1536 (15.0%) DE mRNAs (blue module), 51 (2.8%) DE circRNAs and 1319 (12.9%) DE mRNAs (brown module), and 0 (0.0%) DE circRNAs and 119 (1.2%) DE mRNAs (yellow module) (Figure 5A). GO enrichment analysis of the host genes of the DE circRNAs and their corresponding DE mRNAs assigned in each module revealed that a wide range of biological processes were perturbed during MERS-CoV infection (Figures 5B and 5C). For example, in the turquoise module which comprised the largest number of DE circRNAs and DE mRNAs, the most perturbed processes included DNA-dependent transcription (GO:0006351), regulation of transcription (GO:0006355), protein phosphorylation (GO:0006468) and dephosphorylation (GO:0006470), and protein ubiquitination involved in ubiquitin-dependent protein catabolic process (GO:0042787) (*P* < 0.05).

### RNA transcripts with potentially important roles in MERS-CoV infection identified in a comprehensive circRNA-miRNA-mRNA network

**Figure 6.**
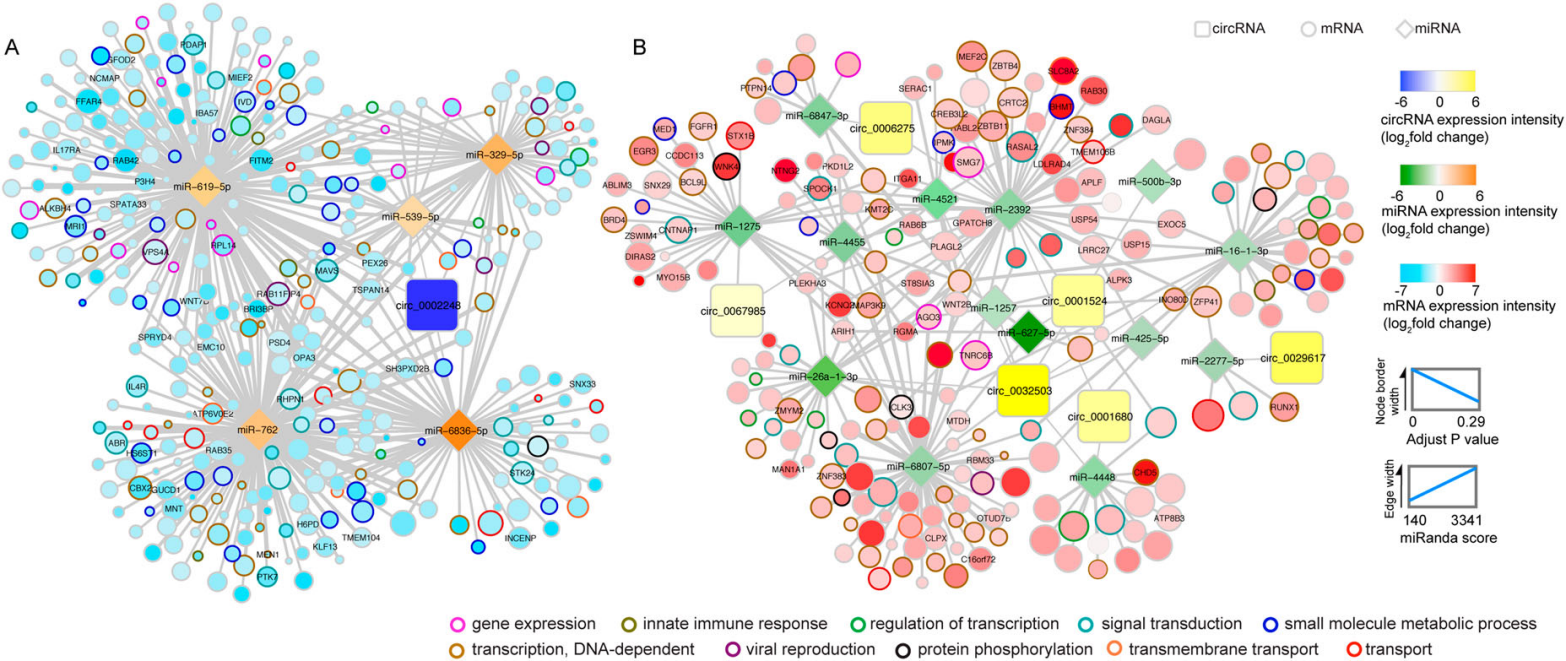
**Potential viral pathogenic circRNAs in the ceRNA co-regulatory network.** circRNAs, miRNAs, and mRNAs potentially involved in MERS-CoV pathogenesis were represented by triangle, rectangle, and circle nodes, respectively. The border thickness and filling colour of each node were mapped according to the expression and adjust *P* value of each RNA at 24 h post MERS-CoV infection. The size of mRNAs was proportional to their correlation extent with selected circRNAs. The stronger that correlation, the larger the node. Top 10 overrepresented GO terms were adopted to colour the border of mRNAs identified in the interactome, and the mRNAs strongly correlated with miRNAs (r < −0.85, cP < 0.05) and circRNAs (r > 0.85, cP < 0.05) simultaneously were labelled with name. Edge thickness was proportionally correlated with the predicted interaction between each circRNA-miRNA pair and miRNA-mRNA pair as defined by miRanda. Among the 6 circRNAs which were significantly upregulated, circ_0006275 and circ_0067985 were selected for further validation. The expression levels of circ_0032503, and the target miRNA of circ_0001680 and circ_0001524 were low, and were therefore not suitable for siRNA knockdown experiment. circ_0029617 only interacted with 1 miRNA and was therefore not selected for further validation.

A comprehensive network showing the interactions among the identified DE circRNAs, DE miRNAs, and DE mRNAs would provide important insights into the potential roles of these RNAs in MERS-CoV infection. To construct this circRNA-miRNA-mRNA network, we included the circRNA-miRNA pairs and miRNA-mRNA pairs with strong negative correlation (r < −0.7 and c*P* < 0.05), and the circRNA-mRNA pairs with strong positive correlation (r > 0.7 and c*P* < 0.05). As showed in Figure 6, the circRNA-miRNA-mRNA network comprised 7 DE circRNAs (upregulated: hsa_circ_0001524, hsa_circ_0001680, hsa_circ_0006275, hsa_circ_0029617, hsa_circ_0032503, and hsa_circ_0067985; downregulated: hsa_circ_0002248), 19 DE miRNAs (upregulated: miR-16-1-3p, miR-26a-1-3p, miR-425-5p, miR-500b-5p, miR-627-5p, miR-1257, miR-1275, miR-2277-5p, miR-2392, miR-4448, miR-4455, miR-4521, miR-6807-5p, and miR-6847-3p; downregulated: miR-329-5p, miR-539-5p, miR-619-5p, miR-762, and miR-6836-5p), and 547 DE mRNAs (Figure 6 and Supplementary Table 3). A total of 21 circRNA-miRNA pairs, 725 miRNA-mRNA pairs, and 642 circRNA-mRNA pairs were identified. A wide range of biological processes were perturbed in this circRNA-miRNA-mRNA network, with the top 10 being DNA-dependent transcription (GO:0006351), signal transduction (GO:0007165), small molecule metabolic process (GO:0044281), viral reproduction (GO:0016032), innate immune response (GO:0045087), gene expression (GO:0010467), DNA-dependent regulation of transcription (GO:0006355), transmembrane transport (GO:0055085), transport (GO:0006810), and protein phosphorylation (GO:0006468). These results highlighted the impact of MERS-CoV infection on host gene expression.

### circRNAs identified in the circRNA-miRNA-mRNA network significantly modulated target mRNA expression and MERS-CoV replication

**Figure 7.**
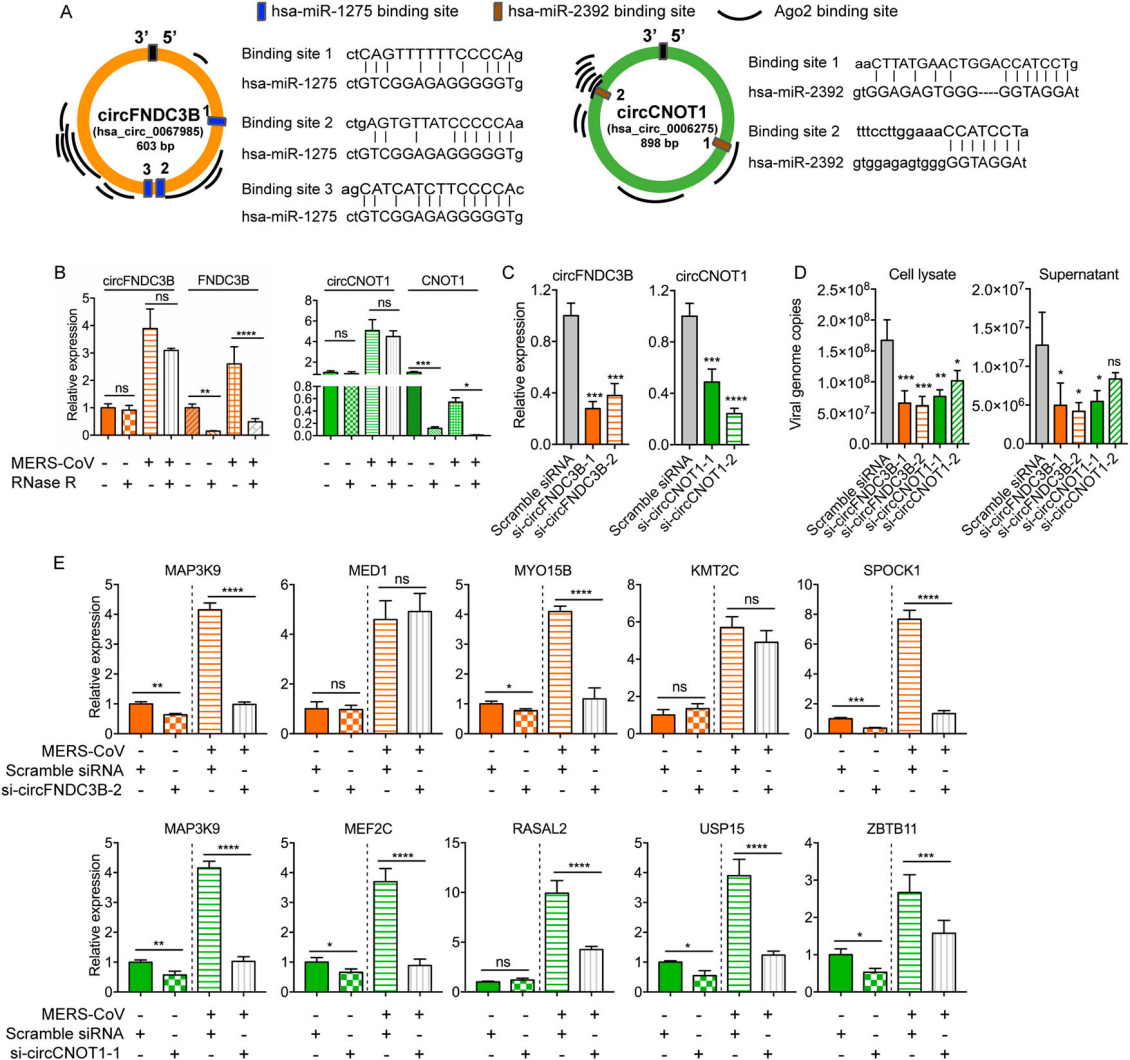
**Inhibitory effect of circFNDC3B and circCNOT1 knockdown on MERS-CoV replication.** (A) The Ago2 protein and miRNA binding sites of circFNDC3B and circCNOT1 predicted by CircInteractome and miRanda, respectively. (B) qPCR validating the expression and RNase R resistance property of the circRNAs circFNDC3B and circCNOT1, and the mRNAs FNDC3B and CNOT1. (C) qRT-PCR examining the knockdown effect of siRNA candidates. Linear RNA of GAPDH was used as internal reference for normalization. *P-value < 0.05; **P-value < 0.01; ***P-value < 0.001; ****P-value < 0.0001, one-way ANOVA. (D) Depletion of circFNDC3B and circCNOT1 suppressed MERS-CoV replication in cell lysate and supernatant. Scramble siRNA was served as a negative control. *P-value < 0.05; **P-value < 0.01; ***P-value < 0.001; ****P-value < 0.0001, one-way ANOVA. (E) CircFNDC3B and circCNOT1 knockdown decreased the expression of representative targeting genes. Student’s t-test was adopted to calculate the significance of gene expression with or without MERS-CoV infection. *P-value < 0.05; **P-value < 0.01; ***P-value < 0.001; ****P-value < 0.0001.

Based on these *in silico* results, we postulated that the identified DE circRNAs in this circRNA-miRNA-mRNA network might play important roles in MERS-CoV propagation and pathogenesis. Therefore, we selected two candidate circRNAs to conduct *in vitro* validation of their functions as miRNA sponges, namely, hsa_circ_0067985 (derived from gene FNDC3B, termed as circFNDC3B) and hsa_circ_0006275 (derived from gene CNOT1, termed as circCNOT1). They were chosen as they were significantly upregulated, had relatively high baseline expression in mock-infected cells, interacted with multiple miRNAs, and had multiple putative Argonaute 2 (Ago2) binding sites (Figure 7A).

Ago2 protein is a core component of RNA-induced silencing complex that binds miRNAs to target mRNAs [47]. Explorations on the RBPs that could bind to these circRNAs through CircInteractome showed that both circFNDC3B and circCNOT1 contained multiple binding sites for Ago2 protein and supported our postulation that these circRNAs could act as miRNA sponges (Figure 7A) [32]. We designed divergent qRT-PCR primers and performed RNase R resistance experiments to examine the expression of these circRNAs with or without MERS-CoV infection (Supplementary Table 1). As shown in Figure 7B, no significant reduction in the relative expression of the two circRNAs was detected after RNase R treatment, while the expression of the mRNAs were greatly affected. To examine the biological relevance of these circRNAs during MERS-CoV infection, siRNAs targeting the back-splicing site of circFNDC3B and circCNOT1 were synthesized and transfected into Calu-3 cells to assess whether depletion of these two candidate circRNAs could limit virus replication (Supplementary Table 1). Our results confirmed that our siRNAs could efficiently knock down the circRNA expression (Figure 7C) but not the linear form (Supplementary Figure 2), and most of the tested siRNAs significantly reduced MERS-CoV (but not SARS-CoV or influenza A/H1N1 viruses) replication in both cell lysate and culture supernatant (Figure 7D and Supplementary Figure 3). Importantly, the siRNAs did not affect cell viability (Supplementary Figure 4). Upon successful knock down (Supplementary Figure 5A), the viral load reduction by si-circFNDC3 and si-circCNOT1 were similarly observed in HFL cells (Supplementary Figure 5B). Moreover, over-expression of circFNDC3B and circCNOT1 in Calu-3 cells enhanced virus replication (Supplementary Figure 6). To validate our hypothesis that circFNDC3B and circCNOT1 function by sequestering their target miRNAs to regulate mRNA expression, we evaluated the expression of their representative target mRNAs after knockdown with si-circFNDC3B-2 or si-circCNOT1-1 which were selected based on their higher inhibitory activity of MERS-CoV replication over si-circFNDC3B-1 or si-circCNOT1-2, respectively. As shown in Figure 7E, circFNDC3B depletion significantly reduced the expression of its representative target mRNAs, including MAP3K9, MYO15B, and SPOCK1. Similarly, circCNOT1 depletion reduced the expression of its target mRNAs, including MAP3K9, MEF2C, USP15, and ZBTB11. Taken together, these results supported our hypothesis that the circRNAs identified in our circRNA-miRNA-mRNA network significantly impacted MERS-CoV replication.

## Discussion

As an integral component of the ceRNA network, circRNAs harbouring MREs can regulate gene expression by functioning as miRNA sponges to release the inhibitory effects of miRNAs on target genes [6,7]. The sponging effect of circRNAs has been shown to be more efficient than that of the linear miRNA and lncRNA transcripts [29,48]. It has previously been shown that various DNA and RNA viruses (herpesviruses, polyomaviruses, retroviruses, and hepaciviruses) could modulate viral replication via miRNA-mediated gene regulation [49,50]. In contrast, the role of circRNA in viral replication is less well understood. For coronaviruses, only two circRNA profile analyses on the animal-pathogenic PEDV and TGEV have been reported [13,15]. In this study, we used the highly human-pathogenic MERS-CoV as a model to demonstrate the interactions of circRNAs with other major components of the host cell ceRNA network and validated the effects of these circRNAs on coronavirus replication.

In our comprehensive profiling of the circRNAs, miRNAs, and mRNAs in human lung epithelial cells with or without MERS-CoV infection, a number of important observations were made. First, a large number of circRNAs (49337) were predicted, including 16285 (33.0%) previously annotated circRNAs and 33052 (67.0%) novel putative circRNAs. Most of these circRNAs were derived from coding regions (83.0%). This is in line with the findings from PEDV-infected porcine intestinal epithelial (IPEC-J2) cells [13], and may represent a conserved character of coronavirus-infected cells. Second, most (84.5%) of these putative circRNAs had low expression levels as evidenced by their small number (1–5) of supported reads. This finding corroborated with those identified in PEDV-infected porcine intestinal epithelial cells, avian leukosis virus subgroup-J-infected chicken hepatic cells, and simian vacuolating virus 40-infected Vero cells [8,10,13]. Third, the mean GC content percentage of the circRNAs was significantly higher than those of the miRNAs and mRNAs. As the GC content is positively correlated with the stability of RNA transcripts, our finding corroborated with the consensus that circRNAs are usually highly stable [29,38]. Finally, our data showed that the number of different circRNAs generated from individual host genes was highly variable, suggesting a differential potential of gene regulation by diverse host genes through circRNA generation.

Our comprehensive circRNA-miRNA-mRNA network showed that MERS-CoV induced significant changes in the expression of many host cell circRNAs, miRNAs, and mRNAs. Compared with the circRNA-miRNA-mRNA network in Madin-Darby Canine Kidney (MDCK) cells infected with influenza A/H3N2, our network in MERS-CoV-infected Calu-3 cells demonstrated a higher number of DE circRNAs (7 vs 3), miRNAs (19 vs 1), and mRNAs (547 vs 9). This might imply that MERS-CoV infection induced a more profound and global change in the host ceRNA network compared to the less virulent influenza A/H3N2, although the differences in experimental set up should also be considered [14]. The DE circRNAs in our network were associated with a wide range of biological, cellular, and molecular processes. Interestingly, using both KEGG pathway and GO functional analyses, we showed that ubiquitin-mediated proteolysis was significantly perturbed in MERS-CoV infection. The papain-like protease of MERS-CoV exhibits deubiquitinating activity and is involved in proteolysis of the viral polyprotein during virus replication [51,52]. Inhibitors of MERS-CoV papain-like protease such as 6-mercaptopurine and 6-thioguanine exhibit antiviral activity *in vitro* [18]. Modulation of the circRNAs associated with ubiquitin-mediated proteolysis identified in our study may provide a new antiviral strategy for MERS-CoV infection.

To validate the biological relevance of the DE circRNAs identified in our network, we selected two DE circRNAs and investigated their effects on MERS-CoV replication and the expression of their target genes in human lung epithelial cells with or without siRNA knockdown. Our results showed that specific knockdown of circFNDC3B and circCNOT1 significantly reduced MERS-CoV viral load in both Calu-3 and HFL cells, which was potentially associated with the downregulation of circFNDC3B- and circCNOT1-regulated target genes. For example, MAP3K9 is an upstream modulator of the mitogen-activated protein kinase (MAPK) pathways which influences many aspects of cell proliferation, migration, and apoptosis [53]. The extracellular signal-regulated kinase (ERK)/MAPK signalling response is specifically modulated in MERS-CoV infection [54]. In this regard, we showed that siRNA knockdown of either circFND3B or circCNOT1 resulted in significantly reduced expression of MAP3K9 and provided novel insights to modulate the ERK/MAPK pathway as a host-targeting antiviral strategy for MERS-CoV infection.

In addition to MAP3K9, siRNA knockdown of circFNDC3B or circCNOT1 similarly resulted in significantly reduced expression of other target genes. Ubiquitin-specific protease 15 (USP15) promotes RIG-I-mediated antiviral signalling by interacting with ubiquitin E3 ligase tripartite motif protein 25 (TRIM25) and plays important roles in the replication of various DNA and RNA viruses, including human papillomaviruse (HPV), human immunodeficiency virus (HIV), and hepatitis C virus (HCV) [55–58]. Myocyte enhancer factor 2C (MEF2C) is a transcription factor that is associated with the super-enhancer activity and cancer progression of Epstein–Barr virus infection [59]. The functions of SPARC/Osteonectin, Cwcv and Kazal-like Domains Proteoglycan 1 (SPOCK1), Zinc Finger and BTB Domain Containing 11 (ZBTB11), and myosin XVB (MYO15B) in viral infection are unclear.

In summary, our study provided novel insights into the ceRNA network perturbations and biological relevance of circRNAs in MERS-CoV infection. Knockdown of specific DE circRNAs in MERS-CoV infection resulted in significantly reduced viral load and may pave new ways for host-targeting antiviral strategies for this highly virulent emerging virus.

## Supplementary Material

Supplemental Material
