# Supplementary material for: Competing endogenous RNA network profiling reveals novel host dependency factors required for MERS-CoV propagation
Source: Emerg Microbes Infect. 2020 Mar 30;9(1):733–46. doi: 10.1080/22221751.2020.1738277 (PMC7170352; doi:10.1080/22221751.2020.1738277)
Supplement: Supplemental Material [file TEMI_A_1738277_SM5341.zip › SUPPLEMENTARY_MATERIALS_2020_02_25.docx]

**SUPPLEMENTARY MATERIALS**

**Supplementary Figure 1.** GO annotations of genes that could generate more than 30 circRNA candidates.

**Supplementary Figure 2.** siRNAs targeting the circRNAs circFNDC3B and circCNOT1 did not significantly affect the expression of the mRNAs FNDC3B and CNOT1 in Calu-3 cells. Abbreviation: ns, not significant.

**Supplementary Figure 3.** (A) Knockdown of circFNDC3B and circCNOT1 did not significantly affect the replication of SARS-CoV and (B) influenza A/H1N1 virus. Calu-3 cells transfected with siRNAs were challenged with SARS-CoV (MOI = 1.0) or influenza A/H1N1 virus (MOI = 0.1), respectively. Scramble siRNA was included as negative control. At 24 hpi, the cell lysate was harvested for viral genome copy measurement. Abbreviation: ns, not significant.

**Supplementary Figure 4.** Depletion of circFNDC3B and circCNOT1 did not significantly affect the cell viability of Calu-3 cells at 24 and 48 hours post-transfection. Abbreviation: ns, not significant.

**Supplementary Figure 5.** (A) siRNAs targeting circFNDC3B and circCNOT1 significantly reduced the expression of circFNDC3B and cirCNOT1 and (B) reduced viral replication in HFL cell lysate and supernatant. **P-value < 0.01; ***P-value < 0.001; ****P-value < 0.0001, one-way ANOVA.

**Supplementary Figure 6.** (A) The expression of circFNDC3B and circCNOT1 in Calu-3 cells after transfection of circFNDC3B and circCNOT1 recombinant plasmids or vector plasmids were detected by qRT-PCR. GAPDH was used as internal reference for normalization. Data represented mean ± standard deviation, n = 3. *P < 0.05 (Student's t-test). (B) Overexpression of circFNDC3B and circCNOT1 enhanced MERS-CoV replication in Calu-3 cell lysate and supernatant. **P-value < 0.01, one-way ANOVA.

**Supplementary Table 1.** Primers and siRNAs used in the study.

**Supplementary Table 2.** List of genes generating ≥30 circRNAs.

**Supplementary Table 3.** circRNAs, miRNAs, and mRNAs with potential roles in the pathogenesis of MERS-CoV infection.
