# Supplementary material for: Competing endogenous RNA network profiling reveals novel host dependency factors required for MERS-CoV propagation
Source: Emerg Microbes Infect. 2020 Mar 30;9(1):733–46. doi: 10.1080/22221751.2020.1738277 (PMC7170352; doi:10.1080/22221751.2020.1738277)
Supplement: Supplemental Material [file TEMI_A_1738277_SM5341.zip › Table S1.docx]

siRNAs

| Name | sense | antisense |
| --- | --- | --- |
| si circCNOT1-1 | AGGACTTGAAGTATTGAAGAA | TTCTTCAATACTTCAAGTCCT |
| si circCNOT1-2 | GGACTTGAAGTATTGAAGAAT | ATTCTTCAATACTTCAAGTCC |
| si circFNDC3B-1 | GACTTGCAAGGACCTGCTGAA | TTCAGCAGGTCCTTGCAAGTC |
| si circFNDC3B-2 | TTGCAAGGACCTGCTGAAGTT | AACTTCAGCAGGTCCTTGCAA |

primers

| Name | Forward primer | Reverse primer |
| --- | --- | --- |
| MERS-CoV NP | CAAAACCTTCCCTAAGAAGGAAAAG | GCTCCTTTGGAGGTTCAGACAT |
| GAPDH | ATTCCACCCATGGCAAATTC | CGCTCCTGGAAGATGGTGAT |
| circCNOT1 (hsa_circ_0006275) | ATGGTACATGAGAGGGGAGCA | GTGACCTCCCGAACACCAAA |
| circFNDC3B (hsa_circ_0067985) | AACAGAGCGACGAGCAAGAA | TAACCTGGAGGCACATGAATGG |
| CNOT1 | CCAGCCCAAAAGTGCTCAAC | GACAAGTCCGTCTGCTGGAA |
| FNDC3B | ACCATTGCTGAACGGAGAGG | GAAAGTTGGGCAATCGCAGG |
| MAP3K9 | GTCTCCCTGACCCCAATGTG | CAAGAAAGTTGGCAAAGACCAGA |
| MED1 | TGGACTGGGCTCTCATCTCA | CTGGGATTGGCTGGGGAAAT |
| MEF2C | CTGCTCGCAGTCACAGACAC | TTTCCTGTACTTGTCCTCAGACTCA |
| MYO15B | CTCCTGAGTGTAGCGCGAG | TCCAAGGAGAGTCGGGGTAG |
| KMT2C | TGCTTACAGCTGCAGGACTC | ACCCGGTAGGACAAATACTGG |
| RASAL2 | TGGCAAAATTGGGGCCTCTC | GAGACGCTCCGACCATTAGG |
| SPOCK1 | GCCATCCATCCACCAACTGA | AGACAGGTACGAGCTGTTGC |
| USP15 | ATGCTGCCGAGGACTTTGAA | AGAAAGTTGGCAAGTTAGTGTG |
| ZBTB11 | ACAGCTGTGTGGCTGACTTT | GCTCAACTCCAAAGTTCATGCTT |
