# Supplementary figures and images for: Competing endogenous RNA network profiling reveals novel host dependency factors required for MERS-CoV propagation
Source: Emerg Microbes Infect. 2020 Mar 30;9(1):733–46. doi: 10.1080/22221751.2020.1738277 (PMC7170352; doi:10.1080/22221751.2020.1738277)

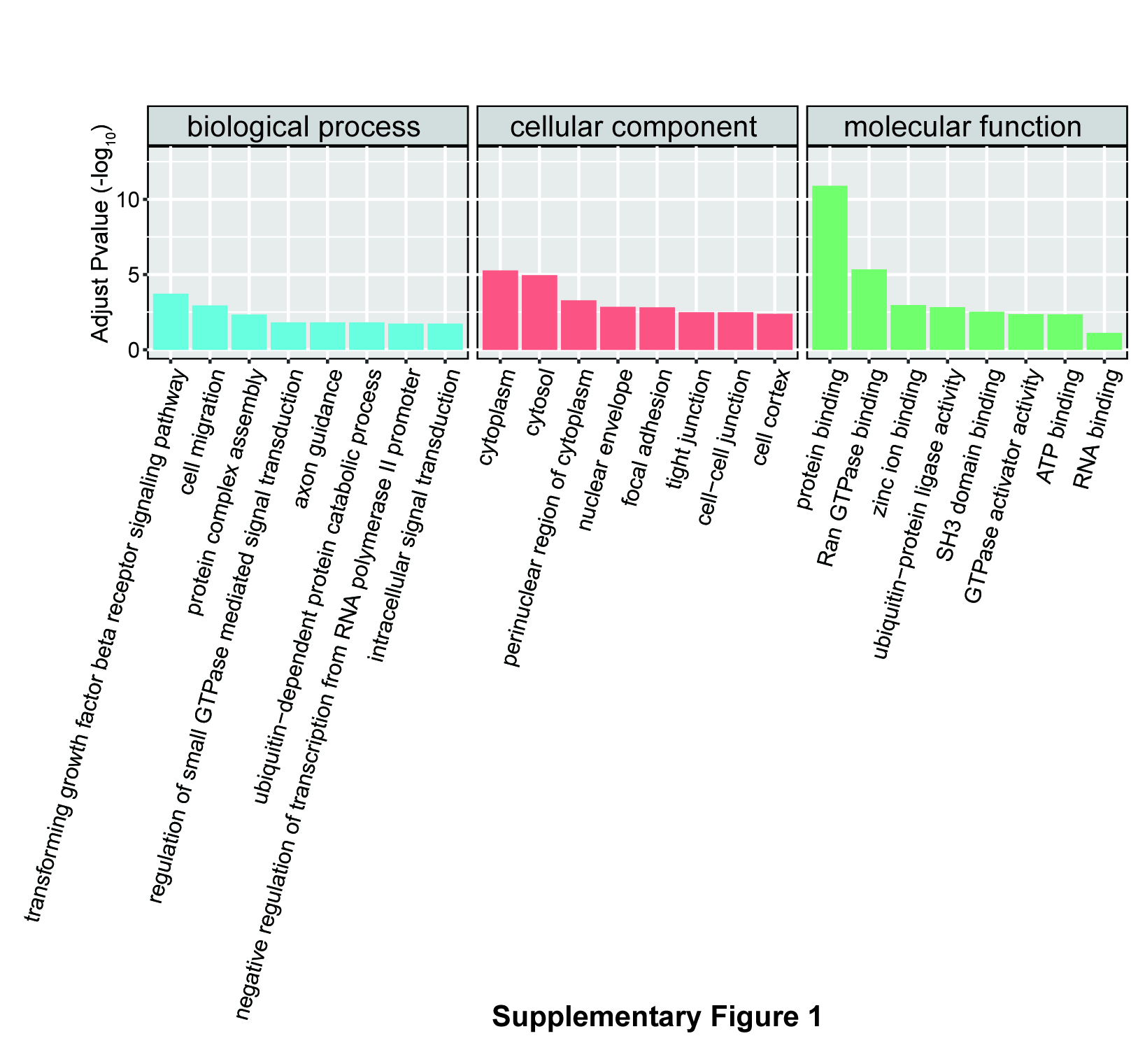

Supplement: Supplemental Material [file TEMI_A_1738277_SM5341.zip › Supplementary Figure 1.tif]

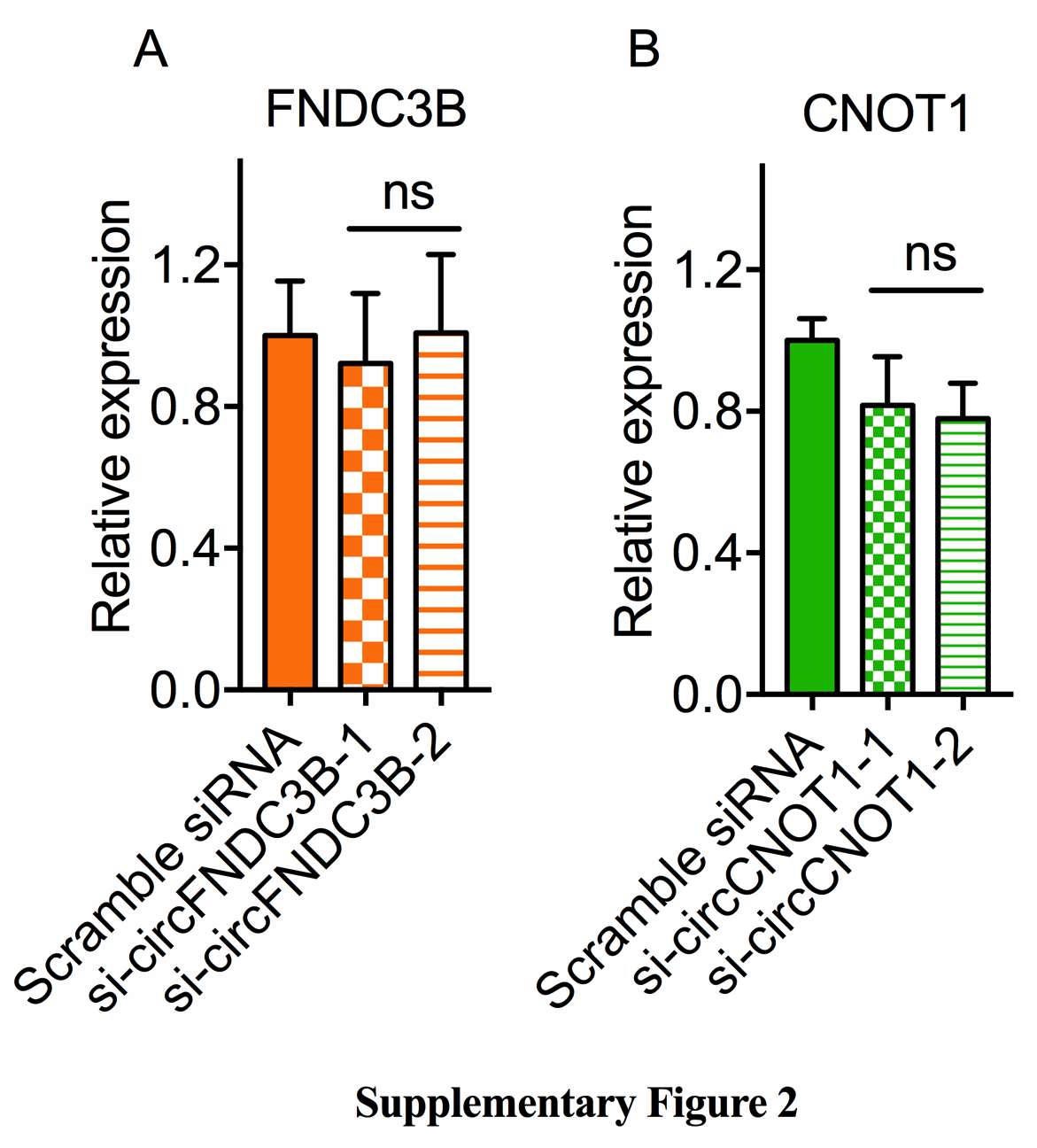

Supplement: Supplemental Material [file TEMI_A_1738277_SM5341.zip › Supplementary Figure 2.tiff]

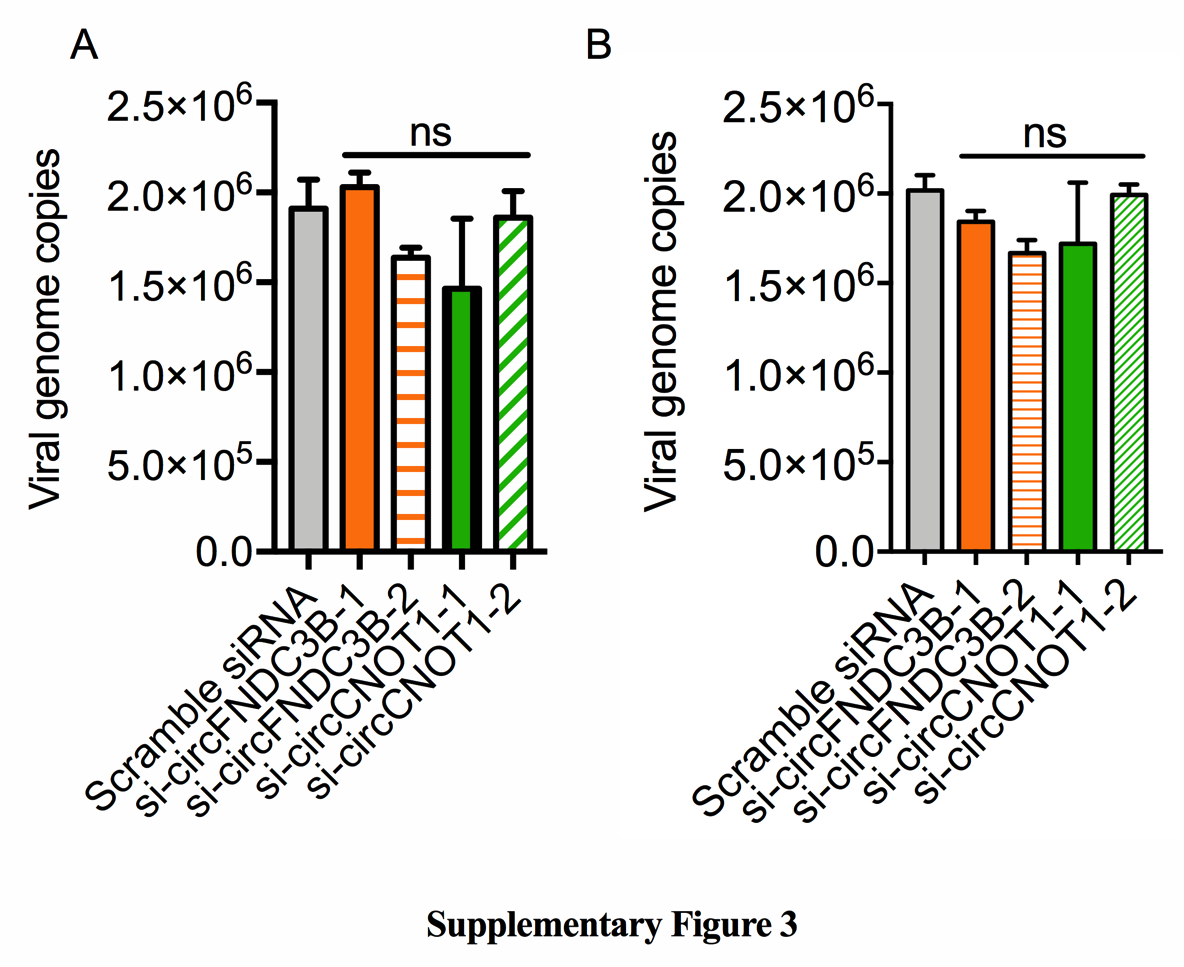

Supplement: Supplemental Material [file TEMI_A_1738277_SM5341.zip › Supplementary Figure 3.tiff]

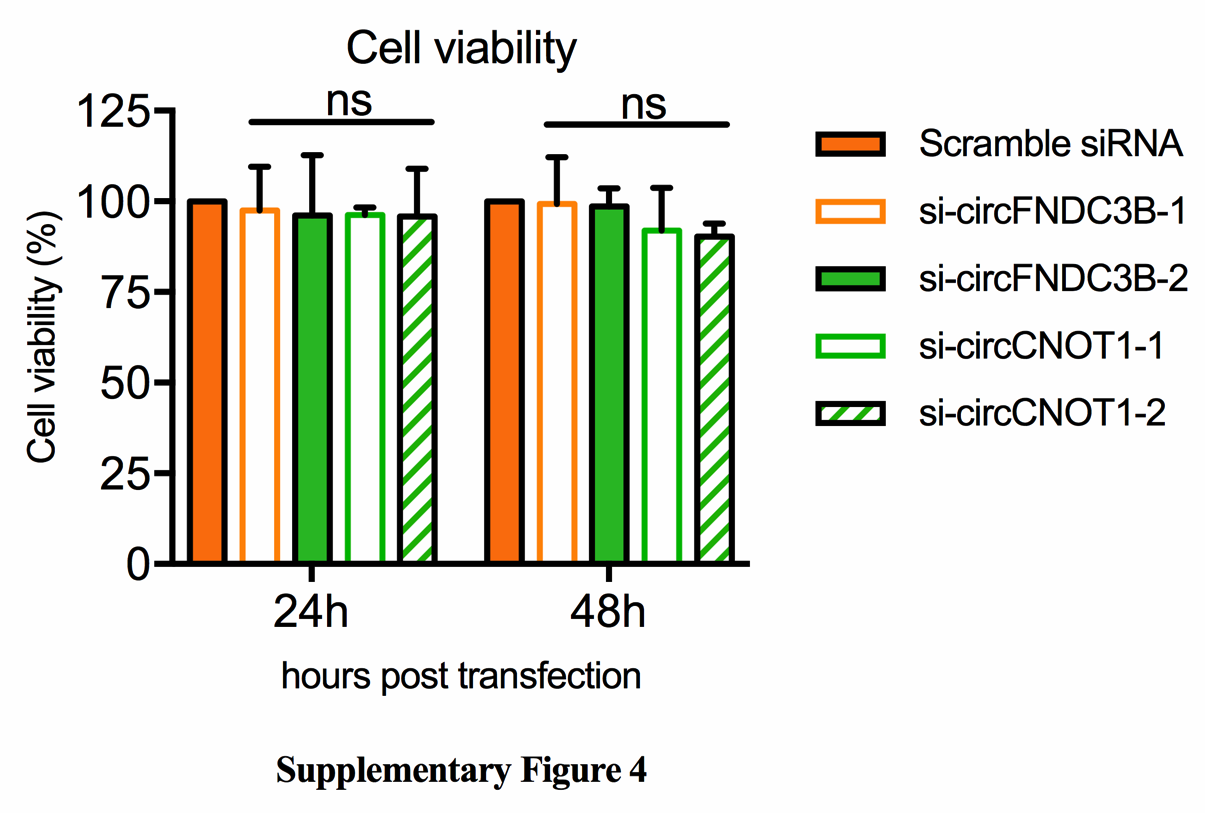

Supplement: Supplemental Material [file TEMI_A_1738277_SM5341.zip › Supplementary Figure 4.tiff]

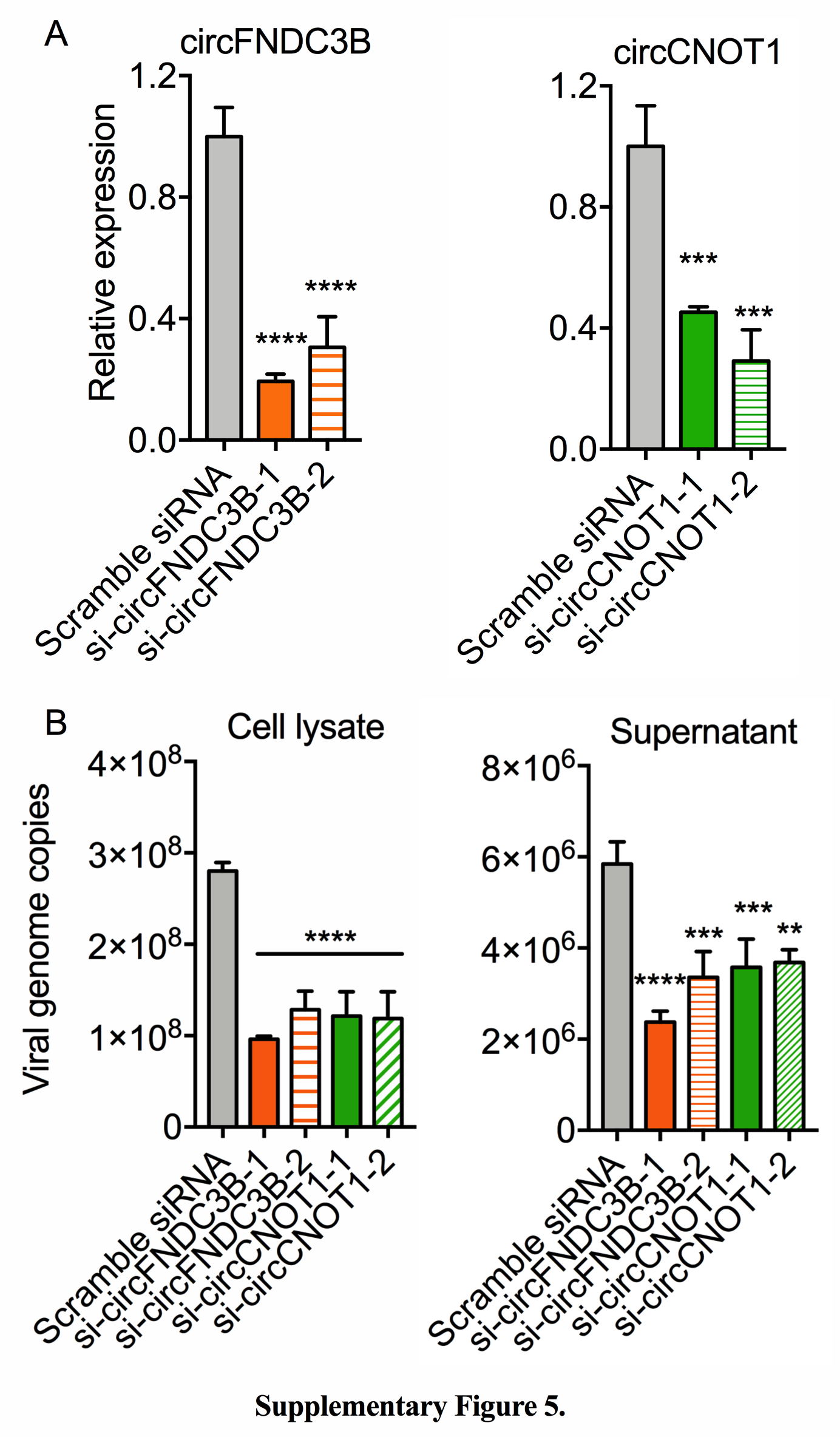

Supplement: Supplemental Material [file TEMI_A_1738277_SM5341.zip › Supplementary Figure 5.tiff]

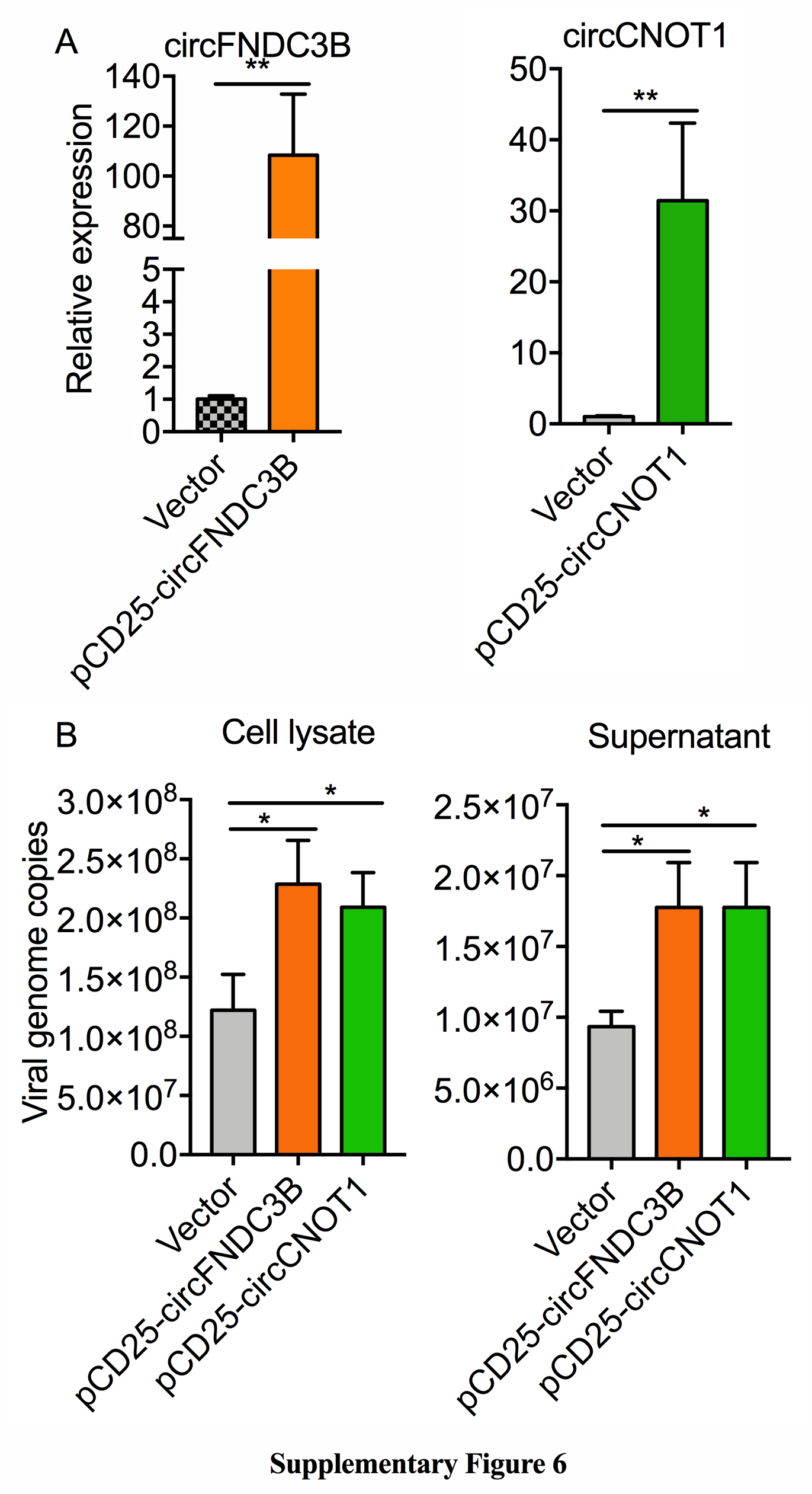

Supplement: Supplemental Material [file TEMI_A_1738277_SM5341.zip › Supplementary Figure 6.tiff]
